# Anti-Phospholipid Antibodies in Patients Undergoing Total Joint Replacement Surgery

**DOI:** 10.1155/2012/142615

**Published:** 2012-10-31

**Authors:** Melissa Simpson, Michael J. Sanfelippo, Adedayo A. Onitilo, James K. Burmester, William Hocking, Steven H. Yale, Joseph J. Mazza

**Affiliations:** ^1^Department of Clinical Research, Marshfield Clinic Research Foundation, 1000 North Oak Avenue, Marshfield, WI 54449, USA; ^2^Departments of Hematology and Oncology, Orthopedic Surgery, Coagulation Laboratory, Marshfield Clinic, 1000 North Oak Avenue, Marshfield, WI 54449, USA

## Abstract

*Background*. Patients undergoing joint replacement remain at increased risk for venous thromboembolism (VTE) compared to other types of surgery, regardless of thromboprophylactic regimen. The pathophysiologic processes rendering this group of patients at risk for VTE are multifactorial. Procedure-specific and patient-specific exposures play a role in the postoperative development of VTE, including the development of anti-phospholipid antibodies (aPL). *Methods*. We measured three aPL (anti-cardiolipin, anti-*β*_2_ glycoprotein, and lupus anticoagulant) in 123 subjects undergoing total knee or hip arthroplasty to describe the presence of these antibodies preoperatively and to describe the rate of postoperative seroconversion among those people who were negative preoperatively. Postoperative antibodies were measured at day 7, 14, and 21. *Results*. The prevalence of aPL antibodies in the preoperative period was 44%, positive subjects were more likely to be smokers (*P* = 0.05) and were less likely to have undergone a previous arthroplasty procedure (*P* = 0.002). Subjects seroconverted in a 21 day postoperative period at a rate of 79%. *Conclusions*. These pilot data suggest that the prevalence of aPL in this population both preoperatively and postoperatively is higher than previously expected. Further studies are needed to describe aPL in a larger population and to establish their clinical significance in populations undergoing joint replacement surgeries.

## 1. Introduction

Despite advances in surgical technique and clinical management of patients undergoing joint replacement surgery, some patients remain at risk for the development of venous thromboembolic events (VTEs). Prior to the standard use of thromboprophylaxis for patients undergoing major orthopedic surgery, the incidence of VTE was as high as 60% and associated with significant mortality and long-term morbidity [1–6]. With routine use of thromboprophylaxis, the incidence of symptomatic VTE and pulmonary embolism (PE) has decreased significantly, and both are now a rare but significant postoperative complication [7–10]. A recently published meta-analysis estimates that about 1 in 100 patients undergoing total knee arthroplasty and 1 in 200 patients undergoing total hip arthroplasty develop a postoperative thrombotic complication in spite of receiving thromboprophylaxis [8].

While the rate of thrombotic complications is much improved compared to rates prior to routine use of thromboprophylaxis, a subset of patients remain at risk for these events. The pathophysiologic processes rendering this subset of patients at high risk for VTE are likely multifactorial and are not fully described. Both procedure-specific and patient-specific exposures play a role in the postoperative development of VTE. Factors contributing to the increased risk of VTE in these patients include procedure-specific exposures such as venous stasis [11], endothelial injury, and tissue inflammation [12]. Patient-specific factors include age, comorbid conditions, and lifestyle factors. Among patient-specific predisposing conditions, the immune response to a surgery of this nature may play an important role in the pathogenesis of VTE.

One potential marker for patients at increased risk for VTE is anti-phospholipid antibodies. 

 aPL are a heterogeneous class of autoantibodies directed against several antigens including (1) phospholipid binding proteins, (2) negatively charged phospholipids alone, or (3) plasma binding protein-phospholipid complexes. These antibodies are broadly divided into anti-cardiolipin antibodies (aCL), anti-beta 2 glycoprotein 1(a*β*_2_GP1), and lupus anticoagulants (LA). Because of their heterogeneity, their importance in thrombogenesis is not completely understood, but they have been associated with venous and arterial thromboembolic complications [13]. 

To date, one study investigated the presence of these antibodies in a population of patients undergoing joint replacement surgery and found that 25% of their patients had abnormal LA and 25% had abnormal aCL. None of their subjects had abnormal a*β*_2_ g [14]. However, it is important to note that this study was limited to subjects with a family or personal history of VTE. To date no studies have been published looking at the incidence of these antibodies in a population of patients that have no prior personal history of VTE and were not selected based on family history.

In order to understand these antibodies in a population of patients undergoing joint arthroplasty, we conducted a pilot study of 123 subjects scheduled for joint replacement surgery and tested for the presence of aPL preoperatively, and at days 7, 14, and 21 postoperatively in a subgroup of study subjects to describe these antibodies in a group of people at increased risk for VTE. 

## 2. Materials and Methods

### 2.1. Study Subjects and Testing Protocol

Study subjects were ≥18 years of age and scheduled for elective unilateral total knee or hip arthroplasty. Exclusion criteria included history of objectively diagnosed deep venous thrombosis or pulmonary embolism, diagnosis of an autoimmune disorder (scleroderma, systemic lupus erythematosus, and rheumatoid arthritis), active malignancy, infection within 30 days of study onset, and residency in a nursing home or long-term care facility at the time of surgery. 

At the time of enrollment, subjects were tested for aCL, a*β*_2_GP1, and LA. One hundred twenty-three participants were screened within 30 days prior to surgery for presence of aPL. Subjects who were aPL negative preoperatively were tested postoperatively for aPL at 7, 14, and 21 days (±2 days). Sixty-nine subjects (56%) were aPL negative preoperatively, complete data was available on 52 (42%) subjects, 5 (4%) subjects completed 2 of 3 follow-up visits, and 12 (10%) subjects did not return for their follow-up appointments. Available data from the 12 subjects who were lost to follow-up and the 5 subjects with incomplete followup are presented.

At the time or preoperative aPL testing, none of the subjects were on anticoagulant medications. All subjects were started on anticoagulant medications ±24 hours of surgery.

The study was approved by the Institutional Review Board and all subjects provided informed consent.

### 2.2. Measurement of Serum Markers

Plasma specimens from all subjects were tested for LA using the dilute Russell's viper venom time (DRVVT) (Siemens Health Care Diagnostics (Deerfield, IL, USA) [15] and the hexagonal phase assay (HPA) Diagnostica Stago, Inc. (Parsippany, NJ, USA) [16] Serum specimens from all subjects were tested for aCL and a*β*_2_GP1 antibodies using a commercial solid phase assay from Inova Diagnostics (San Diego, CA, USA) [17].

DRVVT is reported as the ratio of clotting time without added phospholipid divided by clotting time with phospholipid added. A result of less than 1.3 was considered normal with no evidence of LA. A ratio of 1.3 or greater was considered as evidence of LA. If a subject was positive for DrVVT, we performed a hexagonal phase assay. The hexagonal phase assay was reported as abnormal if an initial prolonged clotting time shortened by ten seconds or more with the addition of phospholipid. Either an abnormal DrVVT result or a hexagonal phase assay were necessary to be considered evidence for LA.

The aCL assay was considered normal if it was less than 15 units for IgG and IgM antibodies and less than 12 units for IgA antibodies. IgG, IgM, and IgA antibodies to *β*_2_GP1 were considered normal if they were less than 20 units and positive if they were over 20 units.

## 3. Statistical Analysis

We present descriptive statistics for all subjects screened preoperatively and for the 69 subjects who were negative aPL negative preoperatively and followed during the postoperative period. The preoperative population was stratified by the presence of aPL. For the description of the subjects followed postoperatively, we compared people who were aPL positive at least twice in the 21-day period following surgery to those who were never positive or positive once.

Chi-squared tests and *t*-tests were used to as appropriate to compare groups. All analyses were performed using SAS 9.2 (Cary, NC, USA).

## 4. Results

Table 1 is a summary of subject characteristics by antibody status preoperatively. The prevalence of aPL antibodies in the preoperative period was 44%. Age, body mass index, gender, and screening for thrombosis did not differ between groups. Subjects who were aPL positive preoperatively were more likely to be smokers (*P* = 0.05) and were less likely to have undergone a previous arthroplasty (*P* = 0.002). The description of antibody positivity among the 54 subjects who were positive preoperatively is shown in Table 2.

Table 3 is a summary of characteristics among subjects who were aPL negative preoperatively, stratified by the development of antibodies postoperatively. The rate of seroconversion in the 21 days following surgery was 79%. Age at time of surgery, mean body mass index, gender, smoking status, total hip replacement (versus total knee replacement), and screening for thrombosis did not differ by aPL status postoperatively. Subjects who developed aPL postoperatively were more likely to have been prescribed warfarin compared to LMWH (41/45 aPL positive versus 0/12 aPL negative; respectively, *P* < 0.0001). Subjects prescribed low molecular weight heparin (LMWH) were aPL positive at a frequency of 4/9 (9%). Positive postoperative aPL was due to LA as shown in Table 4. 

Two subjects (2%) developed a DVT postoperatively. Both of these subjects were positive for LA antibodies preoperatively and were not on anticoagulant medications at that time. In addition, both of these subjects underwent total knee arthroplasty, and this was their first joint arthroplasty. Neither subject was positive for aCL or a*β*_2_G antibodies preoperatively. We do not have data about their antibody status postoperatively.

## 5. Discussion

We identified subjects with a scheduled orthopedic hip or knee arthroplasty and tested them for aPL prior to surgery and after surgery and found that the prevalence of aPL positivity preoperatively was 44% compared to 3 to 10% in the general population [18]. This marked difference in rate may be attributed to the fact that patients undergoing joint replacement surgery tend to be elderly and there is an associated increase in aPL antibodies with advancing age and the medications and diseases that are often concomitant with advancing age. 

Additionally, the incidence of aPL in this study is higher than that found by Bedair et al. [14] in people undergoing joint replacement surgery who were selected because they are at increased risk for VTE. The differences in rates between these two studies suggested that the expression of aPL is independent of existing risk for VTE and possibly heterogeneous in different populations. Further studies are needed to determine what populations (if any) have an associated increased incidence of VTE associated with the presence or development of aPL. Bedair et al. did not report VTE complications in their study.

Although rare, thromboembolic events occur as a result of arthroplasty in spite of aggressive thromboprophylaxis, and these events cause significant morbidity and mortality. The contribution of aPL in this process, if any, remains controversial. Some evidence suggests that positivity is associated with VTE [13], while others found no such association [19, 20]. Further research is needed to determine if there is a subset of patients for whom these antibodies are thrombogenic, if there are subtypes of these antibodies that are more thrombogenic or a combination of these factors.

All subjects who remained antibody negative postoperatively received LMWH, and all of the subjects who were on warfarin developed aPL. Evidence suggests that neither warfarin nor LMWH therapy interferes with the dilute Russell's venom viper time (DRVVT) [17, 21]. 

Perhaps LMWH, by some unknown mechanism, prevents the development of antibodies to protein bound phospholipids and therefore affords some degree of protection from an otherwise thrombogenic complication of orthopedic surgery. Support for a protective effect of heparin is given by recent observation that heparin prevents the development of preeclampsia in mice [22]. Further research into this explanation is needed, including investigating this association in a larger population and elucidation of potential mechanisms. 

Two (2%) study subjects developed deep vein thrombosis within 90 days postoperatively in this cohort; this finding is consistent with current literature [8]. Although no conclusions may be drawn from this study alone, the fact that about 1.5% of the population undergoing joint replacement can be expected to develop VTE is evidence that a subset of patients exists for whom VTE continues to be a concern. Among the risk factors that should be investigated in this group are aPL.

It is important to note that while no one can dispute the increased margin of safety that aggressive anticoagulation has brought to these procedures, thromboprophylaxis is not without risk. Identifying risk strata for VTE may mean that thromboprophylaxis can be tailored accordingly to balance prevention of VTE with the concerns associated with thromboprophylaxis. 

The results of this study into aPL development in subjects undergoing knee or hip arthroplasty are hypothesis-generating. To expand these findings, future studies should investigate a larger population, measure all participants prospectively with prolonged followup, measure aPL later in the postoperative period, and correlate aPL results with clinical outcomes. Elucidation of the association between aPL antibodies and VTE in the context of arthroplasty procedures may help identify a subgroup of patients at high risk for development of VTE who would benefit from more aggressive thromboprophylaxis.

## Figures and Tables

**Table 1:** Description of preoperative characteristics in a population undergoing joint replacement surgery by aPL status preoperatively.

Characteristic	Positive for APLA preoperatively	Negative for APLA preoperatively	*P* value
	54 (44)	69 (56)	
Mean age at the time of surgery (±sd)	60.6 (16.8)	63.2 (9.3)	0.32
Mean body mass index—kg/m^2^ (±sd)	34.2 (7.4)	35.0 (7.3)	0.56
*n* female (%)	30 (56)	42 (61)	0.55
*n* current or past smoker (%)	28 (52)	33 (48)	0.05
*n* missing	16 (30)	8 (12)	
*n* receiving a total hip replacement (%)	16 (30)	18 (26)	0.06
*n* with a prior joint replacement surgery (%)	1 (2)	14 (20)	0.002
*n* with an indication to screen for thrombosis post-operatively (%)	8 (15)	7 (10)	0.43

**Table 2:** Antibody positivity among the 54 study subjects who were positive pre-operatively. Normal font numbers are positive for 1 antibody, bold are positive for 2 antibodies, and italic are positive for 3 antibodies.

	Lupus anticoagulant	Anti-cardiolipin	Anti-*β*_2_ glycoprotein
Lupus anticoagulant	27	**4**	**2**
Anti-cardiolipin	*3 *	11	**3**
Anti-*β*_2_ glycoprotin			4

**Table 3:** Characteristics of the joint replacement population post-operatively.

Characteristic *n* (%)	Positive for APLA at least twice post-operatively	Negative/no data for APLA post-operatively	*P* value
	45 (79)	12 (21)	
Mean age at the time of surgery (±sd)	62.1 (8.1)	62.5 (9.7)	0.37
Mean body mass index—kg/m^2^ (±sd)	34.5 (7.3)	38.3 (7.8)	0.12
*n* female (%)	26 (58)	9 (75)	0.28
*n* current or past smoker (%)	25 (56)	4 (33)	0.11
*n* missing	3 (7)	0 (0)	
*n* receiving a total hip replacement (%)	12 (27)	3 (25)	0.91
*n* with a prior joint replacement surgery (%)	6 (13)	4 (33)	0.11
*n* with an indication to screen for thrombosis (%)	5 (11)	1 (8)	0.78
*n* on Lovenox post-operatively (%)	4 (9)	12 (100)	<0.0001
*n* on Coumadin post-operatively (%)	41 (91)	0 (0)	
Mean days on coumadin post-operatively (±sd)	30.8 (10.5)	N/A	

**Table 4:** Antibody positivity among the study subjects who were positive post-operatively. Normal font numbers are positive for 1 antibody, bold numbers are positive for 2 antibodies, and italic numbers are positive for 3 antibodies.

Day 7 (*n* = 42)			
	Lupus anticoagulant	Anti-cardiolipin	Anti-*β*_2_ glycoprotin
Lupus anticoagulant	40	**1**	**1**
Anti-cardiolipin	*0 *	0	**0**
Anti-*β*_2_ glycoprotin			0

Day 14 (*n* = 41)			

Lupus anticoagulant	39	**1**	**1**
Anti-cardiolipin	*0 *	0	**0**
Anti-*β*_2_ glycoprotin			0

Day 21 (*n* = 45)			

Lupus anticoagulant	44	**1**	**1**
Anti-cardiolipin	*0 *	0	**0**
Anti-*β*_2_ glycoprotin			0
